# Efficient numerical analysis of nonlinear liquid dispersion pattern drops using Taylor wavelet collocation

**DOI:** 10.1038/s41598-025-04698-7

**Published:** 2025-07-05

**Authors:** Mahmoud Abd El-Hady, Mohamed El-Gamel, Yasser Kashwaa

**Affiliations:** Department of Mathematics and Engineering Physics, Faculty of Engineering, Mansoura University, Mansoura, Egypt

**Keywords:** Taylor wavelet, Collocation method, Rosenau–Hyman, Operational matrix, Engineering, Mathematics and computing, Applied mathematics, Computational science

## Abstract

In this article, the Taylor wavelet collocation method (TWCM) is presented for solving the nonlinear Rosenau–Hyman equation, which models nonlinear dispersion in liquid drop formation. TWCM outperforms existing methods, such as the Hermite wavelet and other semi-analytical approaches, by providing higher computational efficiency and accuracy. The differential equation is converted into a system of algebraic equations, solved using the Broyden-Quasi Newton algorithm. Numerical examples demonstrate the reliability and robustness of TWCM in handling nonlinear dispersion patterns. All calculations and visualizations are performed using Matlab, showcasing the method’s effectiveness and advancement in analyzing nonlinear liquid dispersion.

## Introduction

Partial differential equations (PDEs) indeed play a crucial role in engineering, physical and chemical fields. They are regarded as the foundation of many mathematical and physical simulations in real-life applications. PDEs are used in order to describe the behavior of physical phenomena. Hence, PDEs gained pivotal role in research in the last few years in order to find out analytical or numerical solutions of these equations. A summary of the essential techniques related to the approximate solution of various partial differential equations is displayed in Table 1.

**Table 1:** Summary of relevant papers concerning PDE.

PDE	Method	Ref.
Gardner and Harry Dym equation	Quartic B-spline	^1^
Time fractional heat equation	Jacobi collocation method	^2^
Spatiotemporal HIV CD4+T cell model,	Backward Euler and Crank-Nicolson	^3^
Biharmonic Equation	Modified Bi-Quintic B-Spline Collocation	^4^
Fractional Newell-Whitehead-Segel	Chebyshev collocation method	^5^
Time fractional Schrödinger equations	Improved L1-Galerkin spectral method	^6^
Time-fractional Swift-Hohenberg equation	Exponential fitting approach	^7^
$(2 + 1)$ dimensional Kundu-Mukherjee-Naskar	Exp-Function method	^8^
PDE with nonlocal boundary conditions	Sinc collocation approach	^9^
Poisson PDE	Weighted average technique	^10^
Nervous biological fractional FitzHugh-Nagumo	Improved Riccati expansion and B-spline	^11^
Temporal fractional foam drainage equation	Residual power series method (RPSM)	^12^
Nonlinear advection-diffusion model	Finite element method	^13^
Benjamin-Bona-Mahony-Burgers equation	Galerkin finite element method	^14^
Generalized regularized long wave equaton	Radial basis functions and Haar wavelets	^15^
Korteweg-de Vries equation	Scale-3 Haar wavelets, Galerkin finite element	^16,17^

Among these pivotal equations are general third-order Rosenau–Hyman partial differential equations, which can be represented in the following general form

1 $$\begin{aligned} \Xi _{t}+\alpha (\Xi ^{n})_\xi +\beta (\Xi ^{n})_{\xi \xi \xi }=0, \end{aligned}$$

where $\alpha$ and $\beta$ are arbitrary constants with several values. The standard Rosenau–Hyman equation was derived from Eq. (1) by setting $n=2$, $\alpha =-\frac{1}{2}$ and $\beta =-\frac{1}{2}$. This kind of equation has been introduced in many researchers^18,19^. The non linear standard Rosenau–Hyman equation is expressed as follows;

2 $$\begin{aligned} \Xi _{t}=\Xi \Xi _{\xi \xi \xi }+\Xi \Xi _{\xi }+3\Xi _{\xi }\Xi _{\xi \xi }, \end{aligned}$$

for $0\le \xi \le 1$, and $0\le t \le \overline{T}$, with initial and boundary conditions as

3 $$\begin{aligned} \begin{aligned}&\Xi (\xi ,0)=f(\xi ), \quad \quad \quad \quad \quad \quad \quad \quad \quad 0\le \xi \le 1,\\&\Xi (0,t)=p(t),\quad \quad \Xi (1,t)=q(t),\quad \quad \Xi _{\xi }(0,t)=r(t), \quad \quad 0\le t \le \overline{T},\\ \end{aligned} \end{aligned}$$

where $f(\xi )$, *p*(*t*), *q*(*t*) and *r*(*t*) are given continuous functions.

The Rosenau–Hyman equation, developed by Philip Rosenau and James M. Hyman in 1993 as part of their research on compactons^20^, provides a simplified framework for studying nonlinear dispersion in the formation of liquid drop patterns. This equation has a wide range of applications across various fields of physics and engineering, including the dynamics of phase transitions and materials under nonlinear stress conditions, the spread of biological substances, nutrient dispersion in ecological systems, and the dynamics of plasma and astrophysical fluids.

Due to the significance and wide-ranging applications of the Rosenau–Hyman differential equation, numerous investigations have been conducted to develop more efficient and accurate solution methods. A variety of analytical and numerical techniques have emerged in the literature to address this equation. A brief survey of these proposed approaches is summarized in Table 2.

**Table 2:** A concise survey of contemporary approaches for solving the Rosenau–Hyman equation.

Type of solution	Method	Author	Ref.
Analytical	q-Homotopy method	Iyiola et al.	^21^
	Adomian decomposition with Padé approximation	Akg$\ddot{u}$l et al.	^22^
	Variational iteration method	Yulita et al.	^23^
	Hybrid method with RDTM	Derya	^24^
	Perturbation-iteration algorithm (PIA),	Mehmet et al.	^25^
	Residual power series method (RPSM)	Mehmet et al.	^25^
Numerical	Finite difference based on Padé approximations	Rus et al.	^26^
	Self-similar radiation method	Rus et al.	^27^
	Finite difference based on radial basis	Mirzaee et al.	^28^
Spectral techniques	Genocchi wavelets	Cinar et al.	^29^
	Fibonacci wavelets collocation method (FWCM)	Kumbinarasaiah et al.	^30^
	Hermite wavelets collocation method (HWCM)	Kumbinarasaiah et al.	^31^

**Fig. 1:**
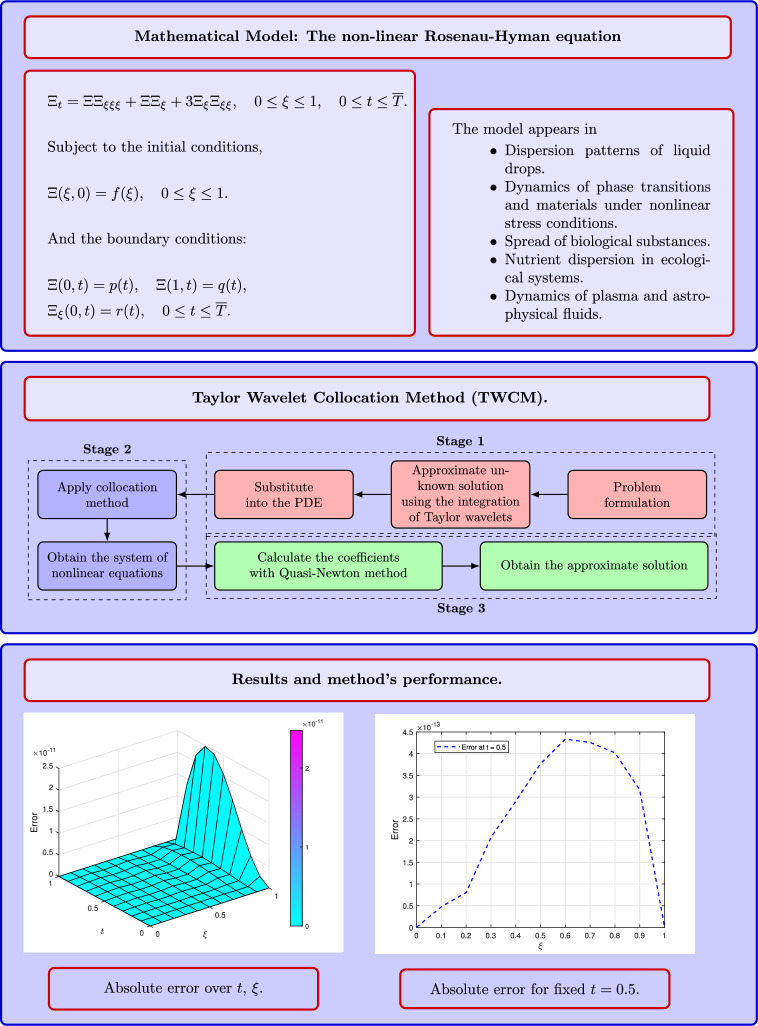
Framework of the Taylor wavelet collocation method for addressing the nonlinear Rosenau–Hyman equation.

The primary aim of this research is to develop a TWCM for the Rosenau–Hyman equation and to assess the robustness and accuracy of the proposed technique through both theoretical and numerical examples. The significant contributions of this study include the introduction of an innovative approach to address non-linearity in the Rosenau–Hyman equation, the execution of comprehensive numerical experiments that demonstrate the effectiveness of the hybrid method, and a comparative analysis with established methodologies to validate the reliability of our approach. Additionally, we present a convergence analysis for function approximation using Taylor wavelets. The framework, workflow, and key findings of this study are illustrated in Fig. 1.

The accurate and efficient analysis of nonlinear liquid dispersion patterns, especially those described by the Rosenau–Hyman equation, is crucial due to its practical significance in various scientific and engineering fields. Applications such as inkjet printing, microfluidics, and coating processes rely heavily on understanding these dispersion dynamics. By effectively modeling liquid drop formation and behavior, we can enhance performance and optimize outcomes in these technologies.

The limitations of existing numerical methods and semi-analytical approaches when applied to the Rosenau–Hyman equation and similar nonlinear dispersive models are significant. These drawbacks include:

- *Lower Computational Efficiency* Many existing methods demand substantial computational resources, leading to longer processing times and increased costs. This inefficiency can hinder their practical application in real-time scenarios.
- *Limited Accuracy* Traditional approaches often struggle to maintain accuracy, particularly when confronted with strong nonlinearities or complex dispersion patterns. This limitation can result in unreliable predictions that compromise the validity of the analysis.
- *Difficulties in Handling Spatial and Temporal Evolution* Existing methods may face challenges in effectively managing the spatial and temporal dynamics of solutions. This can lead to difficulties in accurately capturing the behavior of dispersive phenomena over time.
- *Implementation Complexities* Many numerical techniques involve intricate implementation processes or exhibit convergence issues. These complexities can deter their application in practical settings, as they require specialized knowledge and extensive tuning.

Due to these limitations, we position the TWCM as a novel and advantageous approach designed to address these shortcomings. TWCM promises enhanced computational efficiency and improved accuracy, making it a compelling alternative for analyzing nonlinear liquid dispersion modeled by the Rosenau–Hyman equation.

The collocation method stands out as a prominent spectral technique, offering a versatile framework for approximating solutions to both ordinary and partial differential equations. This approach involves expressing the unknown function as a truncated series of basis functions with undetermined coefficients. These coefficients are then determined by enforcing the differential equation and boundary conditions at a set of collocation points within the solution domain, resulting in a system of algebraic equations. El-Gamel and his research group have made significant contributions to this field over the past decade. They have pioneered novel strategies and techniques, leveraging diverse basis functions such as B-spline^32^, Haar^33^, Sinc^9^, Bernstein^34,35^, Jacobi^36,37^, Bessel^38^ and Chelyshkov^39^ to approximate solutions for differential, integral, and integro-differential equations arising in various engineering applications.

Taylor wavelets are compact, spatially oriented oscillatory functions fundamental to various important spaces. Operational matrices are used to estimate integrals that can transform the problem into a series of algebraic equations with the wavelet basis. TWCM offers several advantages: it provides high accuracy, is quicker to implement, less error components and is simpler and more cost-effective compared to Chebyshev, Haar, Fibonacci, Hermite, Bernoulli, Laguerre and Legendre wavelets. Furthermore, the collocation method utilizing Taylor wavelet is employed to approximate solutions for different equations in engineering and science, such as partial differential equation^40,41^, integral equations^42^, integro-differential equations^43^, fractional order differential equation^44–46^ and system of ordinary differential equations^47,48^.

The rest of the paper is coordinated as follows. Taylor wavelet method is presented in “Preliminaries and relations” section. “Taylor wavelets collocation method for evaluating Rosenau Hyman equation” section presents TWCM for Rosenau–Hyman equation. Convergence of Taylor wavelet method is given in “Convergence analysis of Taylor wavelet” section. Numerical simulations of Rosenau–Hyman problem are depicted in “Simulation examples” section. A brief overview of the results from this fulfillment is discussed in “Conclusions” section.

## Preliminaries and relations

In the next section, we will discuss the fundamental theorems and relationships that will be applied throughout the paper.

### An overview of Taylor wavelets

Taylor wavelets are a class of basis functions recently employed in numerical computations. As polynomial functions, they provide effective approximations for both linear and nonlinear problems. The Taylor wavelets, denoted as $\psi _{n,m}(\xi ) = \psi (k,n,m,\xi )$, are characterized by four parameters: *k*, *m*, *n*, and $\xi$. Here, *k* and *M* can be any integer, while $n = 1, 2, 3, \dots , 2^{k-1}$, and *m* indicates the degree of the Taylor polynomials, where $m = 0, 1, 2, \dots , M-1$. These wavelets are defined over the interval [0, 1) as follows:

4 $$\psi _{{n,m}} (\xi ) = \left\{ {\begin{array}{*{20}l} {2^{{0.5(k - 1)}} \sqrt {2m + 1} \varphi _{m} (2^{{k - 1}} \xi - n + 1),} & {{\text{for}}\;\xi \in \left[\frac{{n - 1}}{{2^{{k - 1}} }},\frac{n}{{2^{{k - 1}} }}\right),} \\ {0,} & {{\text{Otherwise}}} \\ \end{array} } \right.$$

$\varphi _{m}$’s are the Taylor polynomials of degree m which are orthogonal, and $\varphi _{m}=\xi ^{m}$ they build a complete basis over the interval [0, 1]^49^. A function $\Xi (\xi ) \in L_2(\mathbb {R})$ over interval [0, 1] can be expressed as

5 $$\begin{aligned} \Xi (\xi ) =\sum _{n=0}^{\infty }\sum _{m=0}^{\infty }\varrho _{n,m}\psi _{n,m}(\xi ), \end{aligned}$$

where

6 $$\begin{aligned} \varrho _{n,m}=\langle \Xi (\xi ) ,\psi _{n,m}(\xi ) \rangle , \end{aligned}$$

By truncating the series in Eq. (5), we get:

7 $$\begin{aligned} \Xi (\xi ) \approx \sum _{n=1}^{2^{k-1}}\sum _{m=0}^{M-1}\varrho _{n,m}\psi _{n,m}(\xi )=\varvec{\Pi }\,\varvec{\Psi (\xi )}, \end{aligned}$$

where $\varvec{\Pi }$ is the coefficient column vector and the vector of Taylor wavelet function $\psi (\xi )$ are $d=2^{k-1}M$ column vectors.

Here, Eq. (7) can be written again as

8 $$\begin{aligned} \Xi (\xi )\approx \sum _{j=1}^{d}a_{j}\psi _{j}(\xi )=\varvec{\Pi }\,\varvec{\Psi (\xi )}, \end{aligned}$$

where $$\varrho _{j} =\varrho _{n,m}$$, $$\psi _{j}(\xi )=\psi _{n,m}(\xi )$$, $$j=1+m+M(n-1)$$ and

$$\varvec{\Pi }=\left[ \varrho _{1},\varrho _{2},\varrho _{3},...,\varrho _{d}\right] ,\quad \varvec{\psi (\xi )}=\left[ \begin{matrix} \psi _{1}&\psi _{2}&\psi _{3}&\ldots&\psi _{d} \end{matrix} \right] ^{\top }.$$

Let us define the collocation points $\xi _{j}= \frac{2j-1}{2^{k}M}$, $j=1,2, \ldots ,d$, we consider the Taylor wavelet matrix $\phi (\xi )_{d \times d}$ as

9 $$\begin{aligned} \phi (\xi )_{d\times d}=\left[\psi \left(\frac{1}{2d}\right), \psi \left(\frac{3}{2d}\right), \psi \left(\frac{5}{2d}\right), ..., \psi \left(\frac{2d-1}{2d}\right)\right]. \end{aligned}$$

For $k=2$ and $M=4$, then $d=8$ and the Taylor wavelet matrix can be expressed as

$${\phi _{8\times 8}} =\,\left[ \begin{matrix} 1.4142 & 1.4142 & 1.4142 & 1.4142 & 0 & 0 & 0 & 0\\ 0.3062 & 0.9186 & 1.5309 & 2.1433 & 0 & 0 & 0 & 0\\ 0.0494 & 0.4447 & 1.2352& 2.4211 & 0 & 0 & 0 & 0\\ 0.0073 & 0.1973 & 0.9135 & 2.5066 & 0 & 0 & 0 & 0\\ 0 & 0 & 0 & 0& 1.4142 & 1.4142 & 1.4142 & 1.4142\\ 0& 0 & 0 & 0& 0.3062 & 0.9186 & 1.5309 & 2.1433 \\ 0 & 0 & 0 & 0& 0.0494 & 0.4447 & 1.2352& 2.4211 \\ 0 & 0 & 0 & 0& 0.0073 & 0.1973 & 0.9135 & 2.5066\\ \ \end{matrix} \right] .$$

Integrating Eq. (4) yields the following operational matrix of integration of integer order *l*:

10 $$\begin{aligned} I^{l}\psi _{n,m}(\xi )=\left\{ \begin{array}{ll} \,\,0, & \text{ for } \quad \xi <\frac{2n-2}{2^{k}}, \\ \\ \,\,f_{1}, & \text{ for } \quad \ \frac{2n-2}{2^{k}}\le \xi \le \frac{2n}{2^{k}},\\ \\ \,\,f_{2}, & \text{ for } \quad \xi >\frac{2n}{2^{k}}, \\ \\ \end{array} \right. \end{aligned}$$

where

$$\begin{aligned}&f_{1}=\frac{1}{(m+l)!}\left( 2^{\frac{k-1}{2}}\sqrt{2m+1} 2^{m(k-1)}m!\left( \xi -2^{\frac{2n-2}{2^{k}}}\right) ^{l+m}\right) ,\\\\&f_{2}=2^{\frac{k-1}{2}}\sqrt{2m+1} \left( \frac{m!2^{m(k-1)}\left( \xi -\frac{2n-2}{2^{k}}\right) ^{l+m}}{(m+l)!}-\sum _{r=1}^{m} \frac{m!2^{(m-r)(k-1)}\left( \xi -\frac{2n}{2^{k}}\right) ^{l+m-r}}{r!(m+l-r)!}\right) .\\\\ \end{aligned}$$

By substituting collocation points in Eq. (10), we can conclude the operational matrix of integration $P^{l}_{d \times d}=I^{l}\psi _{n,m}(\xi )$. For instance, $k=2$ and $M=4 \Rightarrow d=8$, we have

$${P^{2}_{8\times 8}} =\left[ \begin{matrix} 0.0028 & 0.0249 & 0.0691 & 0.1353 & 0.2210 & 0.3094 & 0.3977 & 0.4861\\ 0.0002 & 0.0054 & 0.0249 & 0.0684 & 0.1403 & 0.2169 & 0.2934 & 0.3700\\ 1.6e-05 & 0.0013 & 0.0101 & 0.0386 & 0.0988 & 0.1647 & 0.2306 & 0.2965\\ 1.4e-06 & 0.0003 & 0.0045 & 0.0240 & 0.0760 & 0.1345 & 0.1929 & 0.2514\\ 0 & 0 & 0 & 0 & 0.0028 & 0.0249 & 0.0691 & 0.1353\\ 0 & 0 & 0 & 0 & 0.0002 & 0.0054 & 0.0249 & 0.0684\\ 0 & 0 & 0 & 0 & 1.6e-05 & 0.0013 & 0.0101 & 0.0386\\ 0 & 0 & 0 & 0 & 1.4e-06 & 0.0003 & 0.0045 & 0.0240\\ \end{matrix} \right] .$$

## Convergence analysis of Taylor wavelet

### Theorem 3.1

*Suppose that the function* $\Xi : [0,1] \rightarrow \mathbb {R}$ *is* $m+1$ *times continuously differentiable and* $\xi \in C^{M+1}\left( \left[ 0,1\right] \right)$. *Let* $S = \text {span}\{\varphi _{0}, \varphi _{1}, \varphi _{2}, \ldots , \varphi _{M-1}\}$ *be a vector space. The approximation function* $\Xi (\xi ) \simeq \sum \limits _{i=0}^{M-1} \varrho _{i} \varphi _{i}(\xi )$ *using Taylor wavelets is the best approximation of* $\Xi$ *out of* *S* *with the mean error bound, which can be presented as follows:*

$$\left\| \Xi - \sum _{i=0}^{M-1} \varrho _{i} \varphi _{i}(\xi )\right\| _{2} \le \frac{\sqrt{2} \,\Gamma \varLambda ^{\frac{2M+3}{2}}}{(M+1)! \sqrt{2M+3}},$$

*where* $$\Gamma = \underset{\xi \in [0,1]}{\max } \left| \Xi ^{(M+1)}(\xi )\right|$$ *and* $$\varLambda =\max \left\{ 1 - \xi _{0}, \xi _{0}\right\}$$.

### Proof

Consider

$$\breve{\Xi }(\xi )= \Xi (\xi _{0})+\Xi ^{'}(\xi _{0})(\xi -\xi _{0})+\frac{1}{2!}\Xi ^{''}(\xi _{0})(\xi -\xi _{0})^{2}+\cdots+\frac{1}{M!}\Xi ^{(M)}(\xi _{0})(\xi -\xi _{0})^{M} .$$

The error can be expressed as:

$$\left| \Xi (\xi ) - \breve{\Xi }(\xi ) \right| = \left| \Xi ^{(M+1)}(\sigma ) \frac{(\xi - \xi _{0})^{M+1}}{(M+1)!} \right| \quad \text {for some } \sigma \in (0,1).$$

Since $\Xi (\xi ) \simeq \sum \limits _{i=0}^{M-1} \varrho _{i} \varphi _{i}(\xi )$ provides the best approximation of $\Xi (\xi )$, we have:

$$\begin{aligned} \left\| \Xi (\xi )-\sum _{i=0}^{M-1} \varrho _{i}\varphi _{i}(\xi ) \right\| _{2}^{2}\le&\left\| \Xi -\breve{\Xi }\right\| _{2}^{2} = \int _{0}^{1} \left[ \Xi (\xi )-\breve{\Xi }(\xi )\right] ^{2} d\xi = \int _{0}^{1} \left[ \Xi ^{(M+1)}(\sigma )\frac{(\xi -\xi _{0})^{M+1}}{(M+1)!}\right] ^2 d\xi , \\\\ \le&\frac{\Gamma ^{2}}{[(m+1)!]^{2}}\int _{0}^{1} \left( \xi -\xi _{0}\right) ^{2M+2}d\xi ,\\\\ \le&\frac{2\Gamma ^{2}\varLambda ^{2M+3}}{\left[ (M+1)!\right] ^{2}(2M+3)}, \\\\ \end{aligned}$$

where $\Gamma =\underset{\sigma \in [0,1]}{\max }\ \left| \Xi ^{(M+1)}(\sigma )\right|$ and $\varLambda =\max \{1-\xi _{0},\xi _{0}\}$. Taking the square root yields the above bound. $\square$

### **Theorem 3.2**

*Suppose that the function* $\Xi$ *satisfy Theorem* 3.1. *Then, the Taylor wavelet approximation* $\breve{\Xi }(\xi )\simeq \sum \limits _{n=1}^{2^{k-1}} \sum \limits _{m=0}^{M-1} \varrho _{n,m}\psi _{n,m}(\xi )$ *approximates* $\Xi (\xi )$ *with mean error bounded as follows:*

$$\Vert \Xi (\xi )-\breve{\Xi }(\xi )\Vert _{2}\le \frac{\sqrt{2}\,\Gamma }{2^{(k-1)(M+1)}(M+1)!\sqrt{2M+3}},$$

### Proof

Let us divide the interval [0,1] into subinterval $I_{k,n}=[\frac{n-1}{2^{k-1}},\frac{n}{2^{k-1}}]$, $n=1,2,...,2^{k-1}$ with the limitation that $\breve{\Xi }(\xi )$ is a polynomial of degree less than $M+1$ that approximates $\Xi (\xi )$ with minimum error. The approximation approaches the exact solution as *k* tends to $\infty$ by using Theorem 3.1, we conclude

$$\begin{aligned} \Vert \Xi (\xi )-\breve{\Xi }(\xi ) \Vert _{2}^{2} =&\sum _{n}\int _{I_{k,n}} \left[ \Xi (\xi )-\breve{\Xi }(\xi )\right] ^{2} d\xi , \\\\ =&\int _{0}^{1} \left[ \Xi (\xi )-\breve{\Xi }(\xi )\right] ^{2} d\xi ,\\\\ =&\sum _{n} \left[ \frac{2\Gamma _{n}(\frac{1}{2^{k-1}})^{\frac{2M+3}{2}}}{(M+1)!\sqrt{2M+3}}\right] ^{2}, \\\\ \le&\frac{2\Gamma ^{2}}{2^{(k-1)(2M+2)}\left[ (M+1)!\right] ^{2}(2M+3)},\\\\ \le&\frac{\sqrt{2}\Gamma }{2^{(k-1)(M+1)}(M+1)!\sqrt{2M+3}}, \end{aligned}$$

where $\Gamma _{n}=\max _{\xi \in I_{k,n}} |\Xi ^{M+1}(\xi )|$. Taking the square root, we get the above bound.

It is clear that the error between exact solution $\Xi (\xi )$ and Taylor wavelet approximation solution $\breve{\Xi }(\xi )$ diminishes with $2^{-(k-1)(M+1)}$. $\square$

### **Theorem 3.3**

^41^ *Any* $\Xi (\xi )=\Xi ^{'''}(\xi ) \in L^{2}(\mathbb {R})$ *is a continuous function defined on [0,1]. If* $\Xi (\xi )$ *is bounded as* $|\Xi ^{'''}(\xi )| \le \beta , \forall \xi \in [0,1]$, *then*

$$\begin{aligned} \varepsilon =&\left\| \Xi (\xi )-\sum \limits _{n=1}^{2^{k-1}} \sum \limits _{m=0}^{M-1} \varrho _{n,m}\psi _{n,m}(\xi )\right\| ^{2}_{2},\\ \le&\,2^{-2k+2}\,\beta ^{2}\,\rho ^{4}\,\sum \limits _{n=2^{k-1}}^{\infty }\,\frac{1}{2n+1}\,\sum \limits _{r=2^{k-1}}^{\infty }\,\frac{1}{2r+1}\, \sum \limits _{m=M}^{\infty }\,\frac{1}{\left( 2m+1\right) ^{2}}\,\sum \limits _{s=M}^{\infty }\,\frac{1}{\left( 2s+1\right) ^{2}}, \end{aligned}$$

*where* $$\rho =\int ^{1}_{-1}\left| \varphi ^{'}_{m+1}(s_{1})ds_{1}\right|$$.

*Here, all four series converge, and as* *k* and *M* *approach to* $\infty$, $\varepsilon$ *approaches zero. Consequently, the method based on Taylor wavelet collocation converges*.

### Theorem 3.4

^41^ *Assume* $\Xi (\xi ,t) \in L^{2}(\mathbb {R} \times \mathbb {R})$ *is a continuous function defined on [0,1)* $\times$*[0,1). If* $\Xi (\xi ,t)$ *is bounded such that* $\left| \Xi (\xi ,t)\right| <\varTheta$, *then Taylor wavelet approximation solution* $\Xi (\xi ,t)=\sum \limits _{n=1}^{\infty }\sum \limits _{m=0}^{\infty }\psi ^{\top }_{m,n}(\xi )\varrho _{m,n}\psi _{m,n}(t)$ *converges uniformly on it as*

$$\left| \varrho _{m,n}\right| =\left| \kappa \times \delta \times 2^{-2k+2}\times \frac{1}{2m+1}\right| \,\varTheta ,$$

*where* $\kappa =\int _{-1}^{1}\varphi _m(s)ds$, $\delta =\int _{-1}^{1}\varphi _m(r)dr$, *k* and *n* *any positive integers.*

*However,* $\sum \limits _{n=1}^{\infty }\sum \limits _{m=0}^{\infty }\varrho _{m,n}$ *is absolutely convergent if* *k* *and* *n* *tends to* $\infty$. *Therefore,*

$$\Xi (\xi ,t)=\sum \limits _{n=1}^{\infty }\sum \limits _{m=0}^{\infty }\psi ^{\top }_{m,n}(\xi )\varrho _{m,n}\psi _{m,n}(t)$$

*is uniformly converges to required solution.*

### Theorem 3.5

^50^ Let $\left\{ \xi _{i} = t_{i} \mid i = 1, 2, \ldots , d \right\}$ *be a set of* $\frac{2i - 1}{2^{k} M}$ *distinct points in* $[0, 1]$, *and let* $\Xi (\xi , t) \in L[0, 1]$, *where* $L[0, 1]$ *is the set of all continuous functions defined on* $[0, 1]$ *and* $\Xi (\xi , t)$ *is the solution of a partial differential equation. Then, there is exactly one linear combination* $\tilde{\phi } (\xi , t)$ *of polynomial-based wavelet functions that satisfies:*

$$\Xi (\xi _{i},t_{i})= \tilde{\phi }(\xi _{i},t_{i})\quad \forall \quad i=1,2,...,d.$$

## Taylor wavelets collocation method for evaluating Rosenau Hyman equation

In this section, we explore the method for solving Rosenau Hyman equation. First, we assume

11 $$\begin{aligned} \frac{\partial ^{4}\Xi }{\partial t \partial \xi ^{3}}\approx \psi (\xi )^{\top }\varrho \psi (t), \end{aligned}$$

where $\varrho =(\Xi _{i,j})_{d\times d}$ is the matrix of unknown coefficients and $\psi (.)$ is the matrix of Taylor wavelet.

By integrating the Eq. (11) with respect to time *t*, we get:

12 $$\begin{aligned} \Xi _{\xi \xi \xi }\approx (\Xi _{\xi \xi \xi })_{t=0}+\psi (\xi )^{\top }UP^{1}(t), \end{aligned}$$

where $$P^{1}(t)=\int _{0}^{t}\psi (s_{1})ds_{1}$$.

Then applying the initial condition to Eq. (13), we conclude

13 $$\begin{aligned} \Xi _{\xi \xi \xi }\approx f^{'''}(\xi )+\psi (\xi )^{T}\varrho P^{1}(t). \end{aligned}$$

By integrating Eq. (13) three times with respect to $\xi$, we have

14 $$\begin{aligned} \begin{aligned} \Xi (\xi ,t)\approx&\int _{0}^{\xi }\int _{0}^{s_{3}}\int _{0}^{s_{2}}\psi ^{\top }(s_{1})ds_{1}ds_{2}ds_{3}\cdot \varrho P^{1}(t)+f(\xi )-f(0)-\xi f^{'}(0)-\frac{\xi ^{2}}{2}f^{''}(0) \\&+\xi \Xi _{\xi }(0,t)+\frac{\xi ^{2}}{2}\Xi _{\xi \xi }(0,t)+\Xi (0,t), \end{aligned} \end{aligned}$$

let us define $\Xi _{\xi \xi }(0,t)=h(t)$ and apply initial conditions $\Xi (0,t)=p(t)$ and $\Xi _{\xi }(0,t)=r(t)$ to Eq. (14), then

15 $$\begin{aligned} \begin{aligned} \Xi (\xi ,t)\approx&\int _{0}^{\xi }\int _{0}^{s_{3}}\int _{0}^{s_{2}}\psi ^{\top }(s_{1})ds_{1}ds_{2}ds_{3}\cdot \varrho P^{1}(t)+f(\xi )-f(0)-\xi f^{'}(0) \\&-\frac{\xi ^{2}}{2}f^{''}(0)+\xi r(t)+\frac{\xi ^{2}}{2}h(t)+p(t). \end{aligned} \end{aligned}$$

In order to get *h*(*t*), apply the boundary condition $\Xi (1,t)=q(t)$ to Eq. (15), then we have

16 $$\begin{aligned} q(t)\approx \int _{0}^{1}\int _{0}^{s_{3}}\int _{0}^{s_{2}}\psi ^{\top }(s_{1})ds_{1}ds_{2}ds_{3}\cdot \varrho P^{1}(t)+f(1)-f(0)-f^{'}(0)+\frac{f^{''}(0)}{2}+r(t)+\frac{h(t)}{2}+p(t). \end{aligned}$$

From Eq. (16), we extract *h*(*t*) as

17 $$\begin{aligned} h(t)\approx 2\left( q(t)-\int _{0}^{1}\int _{0}^{s_{3}}\int _{0}^{s_{2}}\psi ^{\top }(s_{1})ds_{1}ds_{2}ds_{3}\cdot \varrho P^{1}(t)-f(1)+f(0)+f^{'}(0)-\frac{f^{''}(0)}{2}-r(t)-p(t)\right) , \end{aligned}$$

By substituting Eq. (17) in Eq. (15), we obtain

18 $$\begin{aligned} \begin{aligned} \Xi (\xi ,t)\approx &\int _{0}^{\xi }\int _{0}^{s_{3}}\int _{0}^{s_{2}}\psi ^{\top }(s_{1})ds_{1}ds_{2}ds_{3}\cdot \varrho P^{1}(t)+f(\xi )-f(0)-\xi f^{'}(0)-\frac{\xi ^{2}}{2}f^{''}(0)\\&+p(t)+\xi r(t) +\xi ^{2}q(t)-\xi ^{2}\int _{0}^{1}\int _{0}^{s_{3}}\int _{0}^{s_{2}}\psi ^{\top }(s_{1})ds_{1}ds_{2}ds_{3}\cdot \varrho P^{1}(t)\\&-\xi ^{2}\left( f(1)-f(0)-f^{'}(0)+\frac{f^{''}(0)}{2}+r(t)+p(t)\right) . \\ \end{aligned} \end{aligned}$$

Now by differentiating Eq. (18) with respect to $\xi$, we have

19 $$\begin{aligned} \begin{aligned} \Xi _{\xi }\approx &\int _{0}^{\xi }\int _{0}^{s_{2}}\psi ^{T}(s_{1})ds_{1}ds_{2}\cdot UP^{1}(t)+f^{'}(\xi )-f^{'}(0)-\xi f^{''}(0)+r(t) \\&+2\xi q(t)-2\xi \int _{0}^{1}\int _{0}^{s_{3}}\int _{0}^{s_{2}}\psi ^{\top }(s_{1})ds_{1}ds_{2}ds_{3}\cdot \varrho P^{1}(t)\\&-2\xi \left( f(1)-f(0)-f^{'}(0)+\frac{f^{''}(0)}{2}+r(t)+p(t)\right) , \end{aligned} \end{aligned}$$

and

20 $$\begin{aligned} \begin{aligned} \Xi _{\xi \xi }\approx &P^{1}(\xi )^{\top }\varrho P^{1}(t)+f^{''}(\xi )-f^{''}(0)+2 q(t)-2\int _{0}^{1}\int _{0}^{s_{3}}\int _{0}^{s_{2}}\psi ^{\top }(s_{1})ds_{1}ds_{2}ds_{3}\cdot \varrho P^{1}(t)\\&-2\left( f(1)-f(0)-f^{'}(0)+\frac{f^{''}(0)}{2}+r(t)+p(t)\right) . \end{aligned} \end{aligned}$$

Again by differentiating Eq. (18) with respect to *t*,

21 $$\begin{aligned} \begin{aligned} \Xi _{t}\approx &\int _{0}^{\xi }\int _{0}^{s_{3}}\int _{0}^{s_{2}}\psi ^{\top }(s_{1})ds_{1}ds_{2}ds_{3}\cdot \varrho \psi (t)+\frac{d p}{d t}+\xi \frac{d r}{d t}+\\&+\xi ^{2}\left[ \frac{d q}{d t}- \int _{0}^{1}\int _{0}^{s_{3}}\int _{0}^{s_{2}}\psi ^{\top }(s_{1})ds_{1}ds_{2}ds_{3}\cdot \varrho \psi (t)-\frac{d r}{d t}-\frac{d p}{d t}\right] . \end{aligned} \end{aligned}$$

Now by substituting Eqs. (13), (18), (19), (20), Eqs. (20) and (21) into Eq.(2), replacing $\approx$ by $=$, and taking collocation points $\xi _{i}=\frac{2i-1}{d}$ and $t_{j}=\frac{2j-1}{d}$, $i, j = 1, 2,..., d$, we obtain a system of $d \times d$ of nonlinear algebraic equations:

22 $$\begin{aligned} \begin{aligned}&P^{3}(\xi _{i})\varrho \psi (t_{j}) + \dot{p}(t_{j}) + \xi _{i} \dot{r}(t_{j}) + \xi _{i}^{2}\left[ \dot{q}(t_{j}) - P^{3}(1) \varrho \psi (t_{j}) - \dot{r}(t_{j}) - \dot{p}(t_{j})\right] = \\&\left( f^{'''}(\xi _{i}) + \psi (\xi _{i})^{\top } \varrho P^{1}(t_{j})\right) \left( P^{3}(\xi _{i})\varrho P^{1}(t_{j}) + f(\xi _{i}) - f(0) - \xi _{i}f^{'}(0) - \frac{\xi _{i}^{2}}{2}f^{''}(0) + \xi _{i}r(t_{j}) + \frac{\xi _{i}^{2}}{2}h(t_{j}) + p(t_{j})\right) \\&+ \left( P^{3}(\xi _{i})\varrho P^{1}(t_{j}) + f(\xi _{i}) - f(0) - \xi _{i} f^{'}(0) - \frac{\xi _{i}^{2}}{2}f^{''}(0) + \xi _{i}r(t_{j}) + \frac{\xi _{i}^{2}}{2}h(t_{j}) + p(t_{j})\right) \\&\bigg (P^{2}(\xi _{i})\varrho P^{1}(t_{j}) + f^{'}(\xi _{i}) - f^{'}(0) - \xi _{i}f^{''}(0) + r(t_{j}) \\&\qquad \qquad \qquad \qquad \qquad +2\xi _{i}\bigg [q(t_{j})- P^{3}(1)\varrho P^{1}(t_{j}) - f(1) + f(0) + f^{'}(0) - \frac{f^{''}(0)}{2} - r(t_{j}) - p(t_{j})\bigg ]\bigg ) \\&+ 3\bigg (P^{2}(\xi _{i})\varrho P^{1}(t_{j}) + f^{'}(\xi _{i}) - f^{'}(0) - \xi _{i}f^{''}(0) + r(t_{j}) +\\&\qquad \qquad \qquad \qquad \qquad 2\xi _{i}\bigg [q(t_{j}) - P^{3}(1)\varrho P^{1}(t_{j}) - f(1) + f(0) + f^{'}(0) - \frac{f^{''}(0)}{2} - r(t_{j}) - p(t_{j})\bigg ]\bigg ) \\&\left( P^{1}(\xi _{i})^{\top }\varrho P^{1}(t_{j}) + f^{''}(\xi _{i}) - f^{''}(0) + 2\left[ q(t_{j}) - P^{3}(1)\varrho P^{1}(t_{j}) - f(1) + f(0) + f^{'}(0) - \frac{f^{''}(0)}{2} - r(t_{j}) - p(t_{j})\right] \right) , \end{aligned} \end{aligned}$$

where $P^{3}(\xi )=\int _{0}^{\xi }\int _{0}^{s_{3}}\int _{0}^{s_{2}}\psi ^{\top }(s_{1})ds_{1}ds_{2}ds_{3}$ and $P^{2}(\xi )=\int _{0}^{\xi }\int _{0}^{s_{2}}\psi ^{\top }(s_{1})ds_{1}ds_{2}$ are evaluated using Eq. (10). These nonlinear equations can be solved by using Quasi-Newton Broyden’s method^51^ to obtain the values of Taylor wavelet coefficients. Accordingly, we substitute these coefficients in Eq. (18) to obtain the solution $\Xi (\xi ,t)$ of Eq. (2).

**Figure:**
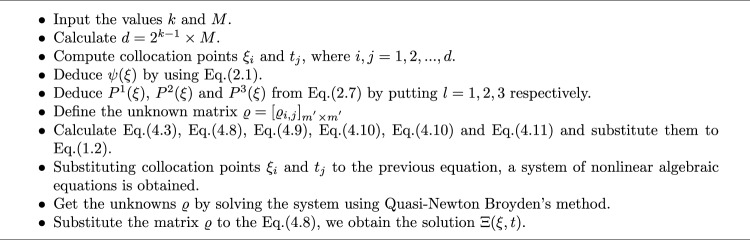
Taylor wavelet algorithm.

### Solution of nonlinear system

The obtained system is transformed into its matrix form with dimension $d\times 1$ as follows:

23 $$\begin{aligned} \varvec{\varpi }(\varvec{\Pi }) = \varvec{0}, \end{aligned}$$

where

- $\varvec{\Pi }$ is the unknowns’ vector.
- $\varvec{\varpi }$ is the equations’ vector.

**Fig. 2:**
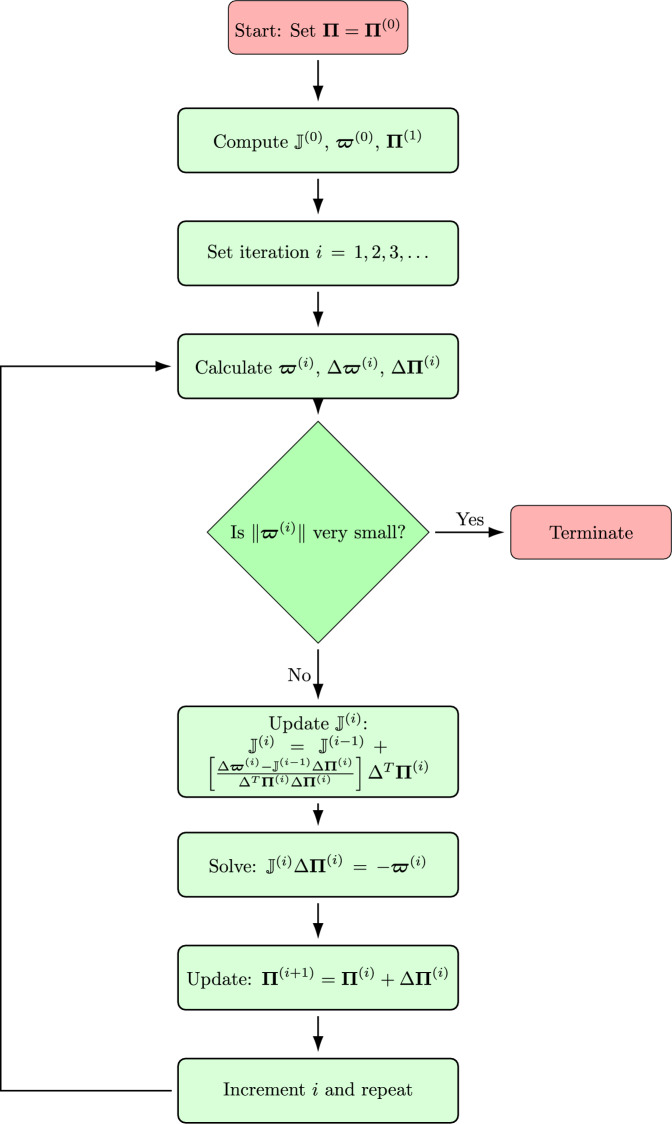
Flow chart of Quasi-Newton Broyden’s algorithm.

This system solved by using quasi-Newton Broyden’s algorithm^36^ for the elements of $\varvec{\Pi }$ and solution $\Xi (\xi ,\eta )$. The graphical steps of this algorithm is explained in Fig. 2, where $\mathbb {J}$ is the well-known Jacobian matrix.

## Simulation examples

In this section, we present illustrative numerical examples to demonstrate the practical application and effectiveness of the TWCM for the nonlinear Rosenau–Hyman equation. These simulations are designed to showcase how TWCM analyzes nonlinear liquid dispersion patterns. The structure of this section will follow the methodological steps previously outlined, illustrating the conversion of the differential equation into an algebraic system and its subsequent solution using the Broyden-Quasi Newton algorithm. The results will highlight the reliability and robustness of TWCM, clearly demonstrating its capabilities in capturing and analyzing nonlinear dispersion phenomena. To assess the efficiency of TWCM, we will solve the nonlinear Rosenau–Hyman equation and compare the obtained results with those from other methods reported in the literature. The simulations were conducted using MATLAB 2024 on a computer with a 2.4 GHz processor and 8 GB of RAM. We will compare our results with those presented in^24,30,31,52^. In this work, $L_{\infty }$ denotes the maximum error, while the total root mean square (RMS) error $L_{RMS}$ of the proposed method is calculated using the formula:

$$\begin{aligned} L_{RMS} = \sqrt{\frac{1}{\hat{N}+1} \cdot \frac{1}{\hat{M}+1} \sum _{i=0}^{\hat{N}} \sum _{j=0}^{\hat{M}} \left[ (\Xi _{\text {exact}})_{i,j} - (\Xi _{\text {numerical}})_{i,j}\right] ^{2}}. \end{aligned}$$

### Example 5.1

Consider the Rosenau–Hyman equation in the form presented in^30,31^:

$$\begin{aligned} \Xi _{t}=\Xi \Xi _{\xi \xi \xi }+\Xi \Xi _{\xi }+3\Xi _{\xi }\Xi _{\xi \xi }\qquad \qquad 0\le \xi \le 1, \qquad t>0, \end{aligned}$$

with initial condition

$$\begin{aligned} \Xi (\xi ,0)=-\frac{8}{3}\cos ^{2}\left( \frac{\xi }{4}\right) , \quad \quad 0\le \xi \le 1, \end{aligned}$$

and boundary conditions

$$\begin{aligned} \Xi (0,t)=-\frac{8}{3}\cos ^{2}\left( \frac{t}{4}\right) ,\quad \Xi (1,t)=-\frac{8}{3}\cos ^{2}\left( \frac{1-t}{4}\right) ,\quad \Xi _{\xi }(0,t)=-\frac{4}{3}\cos \left( \frac{t}{4}\right) \sin \left( \frac{t}{4}\right) , \quad \quad 0\le t \le 1, \end{aligned}$$

the exact solution is

$$\begin{aligned} \Xi (\xi ,t)=-\frac{8}{3}\cos ^{2}\left( \frac{\xi -t}{4}\right) \end{aligned}$$

**Fig. 3:**
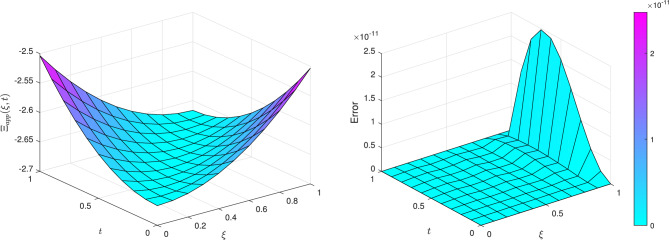
The approximate solution $\Xi (\xi ,t)$ and error for $k=1$ and $M=6$ of Example 5.1.

Figure 3 illustrates the approximate solution $\Xi (\xi ,t)$ on the left and the associated error on the right for $k=1$ and $M=6$, showcasing the solution’s smoothness and continuity, which reflect the method’s ability to capture the underlying physics. The error plot indicates that while some regions exhibit higher error, they remain within acceptable bounds, highlighting the robustness of the numerical approach.

**Fig. 4:**
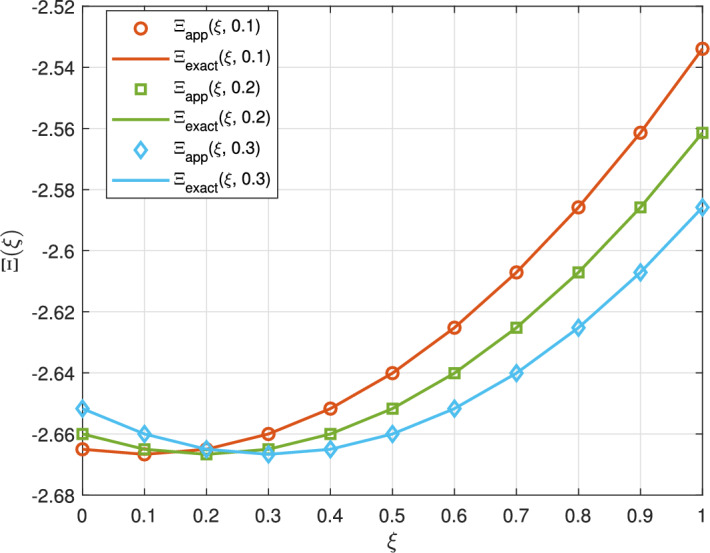
The approximate and exact solution $\Xi (\xi )$ and error for $k=1$ and $M=6$ of Example 5.1 at different *t*.

Figure 4 compares the approximate solution $\Xi (\xi ,t)$ with the exact solution for $k=1$ and $M=6$ at different values of *t*. The close alignment between the approximate solution and the exact solution indicates that the method accurately captures the dynamics of the equation across varying parameters. This close agreement suggests that the proposed method can reliably model the underlying physics of nonlinear phenomena.

**Fig. 5:**
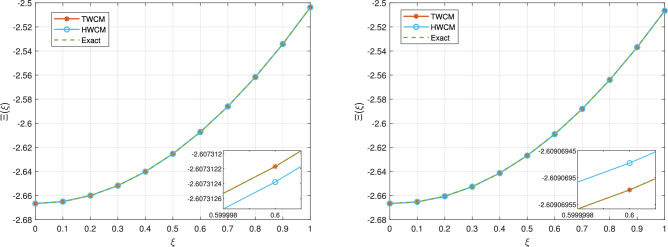
The proposed, Hermite, and exact solutions at $t=0.01$ (left) and $t=0.001$ (right) of Example 5.1.

Figure 5 presents a compelling comparison of the proposed Taylor wavelet collocation method (TWCM) with the Hermite and exact solutions for $t=0.01$ (left) and $t=0.001$ (right), both at $k=2$, and $M=4$. In the left panel, TWCM closely aligns with the exact solution, showcasing its effectiveness in handling the complexities of nonlinear liquid dispersion patterns, with the inset illustrating its precise convergence. The right panel continues this trend, revealing that TWCM maintains a lower error compared to other techniques like HWCM at the finer setting of $t=0.001$. The inset further emphasizes TWCM’s advantages in convergence and accuracy, solidifying its position as a preferred method for solving nonlinear dispersion equations.

The evaluation of the proposed method for solving the nonlinear Rosenau–Hyman equation demonstrates its effectiveness, particularly in comparison to existing techniques. Results presented in Table 3 indicate a low root mean square error (RMS) across various parameter settings, signifying effective convergence to the true solution. Furthermore, the method’s performance relative to other techniques, such as HWCM^31^ and ADM^52^, underscores its superiority in certain cases, suggesting its potential as a valuable tool for researchers dealing with nonlinear differential equations.

**Table 3:** Comparison of the absolute error of example 5.1.

*t*	$\xi =0.1$			$\xi =0.2$		
	TWCM	HWCM	ADM	TWCM	HWCM	ADM
0.1	3.5527e−15	9.9875e−12	2.8908e−11	3.7747e−14	3.9950e−12	2.8809e−11
0.2	1.1102e−14	9.9500e−10	1.8505e−09	5.8619e−14	3.9800e−10	1.8449e−09
0.3	4.8406e−14	9.8877e−10	2.1082e−08	2.5313e−14	3.9551e−10	2.1025e−08
0.4	5.1958e−14	9.8007e−10	1.1846e−07	1.1990e−14	3.9203e−10	1.1818e−07
0.5	4.7517e−14	9.6891e−10	4.5185e−07	8.0380e−14	3.8756e−10	4.5096e−07
	$\xi =0.3$			$\xi =0.4$		
0.1	8.6153e−14	8.9888e−12	2.8639e−11	1.2834e−13	1.5980e−12	2.8398e−11
0.2	1.3500e−13	8.9550e−10	1.8347e−09	1.9495e−13	1.5920e−10	1.8199e−09
0.3	1.9539e−14	8.8989e−10	2.0916e−08	4.4853e−14	1.5820e−10	2.0755e−08
0.4	6.4392e−14	8.8206e−10	1.1761e−07	7.8603e−14	1.5681e−10	1.1674e−07
0.5	2.0738e−13	8.7202e−10	4.4895e−07	2.9043e−13	1.9503e−09	4.4581e−07
	$\xi =0.5$			$\xi =0.6$		
0.1	1.6831e−13	2.4969e−12	2.8085e−11	1.9628e−13	3.5955e−11	$\times$ $\times$ $\times$ $\times$
0.2	2.5179e−13	2.4875e−10	1.8005e−09	2.8865e−13	3.5820e−10	$\times$ $\times$ $\times$ $\times$
0.3	5.9064e−14	2.4719e−09	2.0542e−08	6.8834e−14	3.5596e−09	$\times$ $\times$ $\times$ $\times$
0.4	1.0347e−13	2.4502e−09	1.1559e−07	1.2034e−13	3.5282e−09	$\times$ $\times$ $\times$ $\times$
0.5	3.7703e−13	2.4223e−09	4.4156e−07	4.3343e−13	3.4881e−08	$\times$ $\times$ $\times$ $\times$

Table 4 quantitatively compares the absolute errors among different methods at $t=0.0001$, illustrating that TWCM consistently outperforms the other methods, including FWCM^30^, HWCM^31^, Hybird^24^ and RDTM^24^, in terms of error magnitude. This reinforces the conclusion drawn from the figures regarding the robustness and reliability of TWCM. Moreover, Table 5 presents the root mean square (RMS) and maximum errors using TWCM across varying values of *K* and *M*. The low values for both metrics underline the method’s effectiveness and precision in solving the Rosenau–Hyman equation.

**Table 4:** Comparison of error of Example 5.1 at $t=0.0001$.

$\xi$	TWCM	FWCM^30^	HWCM^31^	Hybird^24^	RDTM^24^
0	0	0	0	0.000250	9.0000e−09
0.1	4.4408e−16	7.4296e−13	9.99e−09	0.000250	1.1114e−05
0.2	4.4408e−16	1.4585e−12	2.00e−08	0.000249	2.2173e−05
0.3	1.3322e−15	8.7761e−11	3.00e−08	0.000248	3.3144e−05
0.4	1.3322e−15	3.2151e−10	4.00e−08	0.000247	4.3981e−05
0.5	1.7763e−15	8.9277e−10	5.00e−08	0.000246	5.4646e−05
0.6	2.2204e−15	2.0746e−09	6.00e−08	0.000244	6.5094e−05
0.7	1.7763e−15	4.2567e−09	7.00e−08	0.000242	7.5284e−05
0.8	1.7763e−15	7.9650e−09	8.00e−08	0.000240	8.5181e−05
0.9	1.3322e−15	1.3882e−08	9.00e−08	0.000237	9.4741e−05
1	0	2.2865e−08	1.00e−07	0.000234	1.0393e−04

**Table 5:** Comparison of the RMS and maximum error using TWCM of Example 5.1.

*K*	*M*	$L_{RMS}$	$L_{\infty }$
1	4	5.4092e−09	2.7987e−08
1	5	9.9500e−10	4.7179e−11
1	6	4.6941e−12	2.4744e−11
2	2	1.1889e−06	5.5411e−06
2	3	2.5005e−09	1.0906e−08
2	4	5.8031e−10	2.8017e−09

Finally, to assess the convergence of the proposed technique, we calculate the computational rate of convergence (ROC) using the following formula:

$$ROC = \frac{\log \frac{\Vert (L_{RMS})_1\Vert }{\Vert (L_{RMS})_2\Vert }}{\log \frac{d_1}{d_2}}.$$

In Table 6, the rate of convergence values are shown, highlighting the speed of convergence of our method. Additionally, the execution time in Table 6 provides a reasonable indication of the efficiency of our proposed scheme.

**Table 6:** A comparison of the rate of convergence and CPU time of TWCM of Example 5.1 for various values of *d*.

*d*	$L_{RMS}$	ROC	CPU time (S)
4	5.4092e−09	–	0.79
5	9.9500e−10	7.59	1.67
6	4.6941e−12	29.38	2.38
8	2.2313e−15	26.59	4.79
10	2.4887e−16	9.83	7.02

### Example 5.2

Consider the Rosenau–Hyman equation as presented in^29^, which can be derived from Eq. (1) by setting $n=3$, $\alpha =-0.1$ and $\beta =1$:

24 $$\begin{aligned} \Xi _t - 0.3\Xi ^{2}\Xi _{\xi } + 6\Xi ^{3}_{\xi }+3\Xi ^{2}\Xi _{\xi \xi \xi }+18\Xi \Xi _{\xi }\Xi _{\xi \xi } = 0, \end{aligned}$$

subject to the initial condition

$$\begin{aligned} \Xi (\xi , 0) = \sqrt{-15c \cosh ^2\left( \frac{\xi \sqrt{0.1}}{3}\right) }, \end{aligned}$$

and the boundary conditions

$$\begin{aligned} \begin{aligned} \Xi (0, t)&= \sqrt{-15c \cosh ^2\left( \frac{-ct\sqrt{0.1}}{3}\right) }, \\ \Xi (1, t)&= \sqrt{-15c \cosh ^2\left( \frac{(1-ct)\sqrt{0.1}}{3}\right) }, \\ \Xi _\xi (0, t)&= -\frac{\sqrt{0.1}}{3} \sqrt{-15c \cosh ^2\left( \frac{ct\sqrt{0.1}}{3}\right) } \tanh \left( \frac{ct\sqrt{0.1}}{3}\right) , \end{aligned} \end{aligned}$$

where *c* is an arbitrary constant and $\Xi = \Xi (\xi , t)$. The exact solution is given by

$$\begin{aligned} \Xi (\xi , t) = \sqrt{-15c \cosh ^2\left( \frac{(\xi -ct)\sqrt{0.1}}{3}\right) }. \end{aligned}$$

**Fig. 6:**
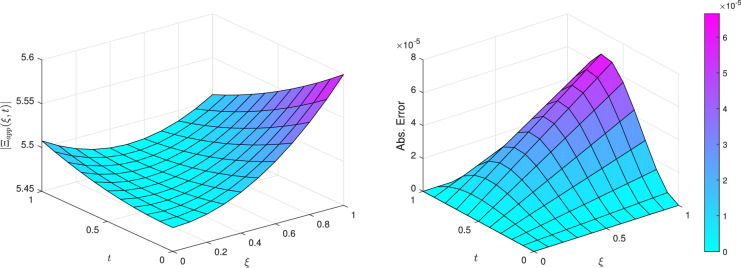
The approximate solution $\left| \Xi (\xi ,t)\right|$ and absolute error for $k=1$ and $M=6$ of Example 5.2.

Applying the solution procedure detailed in Section 4 to Eq.(24) yields the numerical results presented in Table 7 and visualized in Fig. 6.

Table 7 provides a quantitative comparison of the absolute errors between the present method and the Genocchi wavelet collocation method (GWCM)^29^. The results demonstrate that the present method consistently outperforms GWCM in terms of error magnitude. This finding further supports the conclusions drawn from the figures regarding the robustness and reliability of the present method.

**Table 7:** Comparison of absolute error between TWCM and GWCM of Example 5.2 at $c=0.1$.

$(\xi , t)$	TWCM	GWCM
(0.125, 0.125)	8.619e−09	5.544e−09
(0.125, 0.375)	2.586e−08	1.662e−08
(0.125, 0.625)	4.309e−08	2.770e−08
(0.125, 0.875)	6.033e−08	3.879e−08
(0.375, 0.125)	5.542e−08	4.625e−08
(0.375, 0.375)	1.662e−07	1.386e−07
(0.375, 0.625)	2.771e−07	2.309e−07
(0.375, 0.875)	3.879e−07	3.233e−07
(0.625, 0.125)	9.239e−08	1.269e−07
(0.625, 0.375)	2.771e−07	3.792e−07
(0.625, 0.625)	4.619e−07	6.317e−07
(0.625, 0.875)	6.466e−07	8.844e−07
(0.875, 0.125)	6.038e−08	2.484e−07
(0.875, 0.375)	1.811e−07	9.865e−02
(0.875, 0.625)	3.019e−07	9.861e−02
(0.875, 0.875)	4.226e−07	1.724e−06

Figure 6 illustrates the absolute value of the approximate solution $\left| \Xi (\xi , t) \right|$ on the left, alongside the corresponding absolute error on the right, for $k = 1$ and $M = 6$. The smooth and continuous characteristics of the approximate solution reflect the method’s ability to accurately represent the underlying physical phenomena. The error plot reveals that, although some regions display larger errors, these remain within acceptable bounds, underscoring the robustness of the proposed numerical technique. Overall, the results and figures collectively demonstrate that TWCM is a powerful and reliable tool for tackling the complexities associated with nonlinear differential equations, particularly in modeling liquid dispersion patterns.

## Conclusions

In this work, we presented the Taylor wavelet collocation method (TWCM) and applied it to the nonlinear Rosenau–Hyman equation. By utilizing the matrices of the integral operator of the Taylor wavelets, we successfully transformed the partial differential equation into a system of nonlinear algebraic equations. These equations were then solved using the Quasi-Newton Broyden’s method. Our comparative analysis with the Hermite wavelet collocation method (HWCM) and the Adomian Decomposition Method (ADM) demonstrated that the proposed TWCM is more robust and effective in handling the complexities of nonlinear liquid dispersion patterns.

Looking ahead, several promising directions for future research emerge. We aim to explore the application of TWCM to a wider variety of nonlinear equations in fields such as fluid dynamics and materials science, where similar dispersion phenomena occur. Furthermore, we intend to broaden the scope of TWCM by extending its applicability to integro-partial differential equations, which are crucial in modeling phenomena across diverse fields such as aggregation, breakage models, population dynamics, finance, and viscoelasticity.

The Taylor Wavelet Collocation Method’s (TWCM) performance is sensitive to the order of Taylor wavelets (*K*) and the number of collocation points (*d*), where higher values improve accuracy at the cost of computational resources and potential ill-conditioning of the resulting algebraic system. Furthermore, TWCM’s applicability to problems with strong nonlinearities, sharp gradients, or complex domains requires further investigation remains a challenging area for future research. We also intend to conduct comparative studies with a broader range of wavelet methods and semi-analytical techniques. This will help validate the robustness of TWCM across diverse applications. These efforts will not only refine our current approach but also expand its applicability, paving the way for significant advancements in the numerical analysis of nonlinear systems.

In summary, the findings from this study provide a strong foundation for the continued exploration of TWCM and its potential to address complex nonlinear problems in various scientific domains.

## Data Availability

All data generated or analyzed during this study are included in this published article.
